# Quality of life in Vietnamese young adults: A validation analysis of the World Health Organization’s quality of life (WHOQOL-BREF) instrument

**DOI:** 10.3389/fpsyt.2022.968771

**Published:** 2022-12-20

**Authors:** Linh Gia Vu, Long Hoang Nguyen, Cuong Tat Nguyen, Giang Thu Vu, Carl A. Latkin, Roger C. M. Ho, Cyrus S. H. Ho

**Affiliations:** ^1^Institute for Global Health Innovations, Duy Tan University, Da Nang, Vietnam; ^2^Faculty of Medicine, Duy Tan University, Da Nang, Vietnam; ^3^VNU University of Medicine and Pharmacy, Vietnam National University, Hanoi, Vietnam; ^4^National Centre for Youth Substance Use Research, The University of Queensland, Brisbane, QLD, Australia; ^5^Bloomberg School of Public Health, Johns Hopkins University, Baltimore, MD, United States; ^6^Department of Psychological Medicine, Yong Loo Lin School of Medicine, National University of Singapore, Singapore, Singapore; ^7^Institute for Health Innovation and Technology (iHealthtech), National University of Singapore, Singapore, Singapore

**Keywords:** WHOQOL-BREF, validation, reliability, youths, Vietnam

## Abstract

**Background:**

The abbreviated version of the World Health Organization’s Quality of Life (WHOQOL-BREF) instrument has been widely used to assess the quality of life (QOL) of different population groups.

**Aims:**

This study aimed to examine the validity and reliability of the Vietnamese version of WHOQOL-BREF in evaluating the QOL of Vietnamese young adults.

**Methods:**

The WHOQOL-BREF was validated in an online cross-sectional study among 445 young adults from 16 to 35 years in Vietnam. The exploratory factor analysis (EFA) and confirmatory factor analysis (CFA) were performed to examine the factorial structure of the instrument. The reliability and validity of the new factorial model were evaluated.

**Results:**

The EFA and CFA suggested the 3-factor model had better fit models than the theoretical 4-factor model. The internal consistency of factor 1 “External life” and factor 2 “Internal life” were excellent (0.931) and good (0.864), respectively, while the internal consistency of factor 3 “Physical and mental health” was nearly acceptable (0.690). Results indicated that the 3-factor model had good convergent and divergent validity as well as moderate discriminant validity. Scores of factors “External life” and “Internal life” had significant predictive effects on general QOL, general health, and overall QOL (*p* < 0.05). Meanwhile, factor 3 “Physical and mental health” could only predict general health and overall QOL (*p* < 0.05).

**Conclusion:**

This validation study improves understanding of the characteristics of QOL among young adults in Vietnam. While the theoretical model of WHO can be utilized for global comparisons, a new local model should be considered and cross-culturally adapted to successfully capture the progress of public health interventions for promoting young adults’ QOL.

## Introduction

Quality of life (QOL) is defined as a way for people to evaluate their life situation in terms of their objectives, worries, standards, and expectations, as well as the culture and value systems of their community (1). Beyond traditional outcomes like symptoms, morbidity, and mortality, QOL assessment provides insights into multi-dimensions of personal life including physical and psychological states, social relationships, autonomy level, spiritual belief as well as person-environment interaction (1, 2). Self-reported QOL has become an integral component for determining needs and monitoring outcomes of treatment interventions, health service delivery, or health system reform in clinical and public health research (3).

The shifting focus from objective evaluation to subjective QOL assessment has resulted in the proliferation of QOL-related theories and instruments (4–7). The WHOQOL-100 and its shorter form, the World Health Organization’s Quality of Life (WHOQOL-BREF), are commonly used to evaluate people’s QOL under various health circumstances. The WHOQOL-100 instrument was developed in 1995 to inform a comprehensive QOL assessment (8). This tool addresses six areas of life including (1) physical strength, (2) environment, (3) social systems, (4) psychological health, (5) personal/religious/spiritual beliefs, and (6) independence, and its includes 100 entries (8, 9). Despite its comprehensiveness, certain troubles still arose in its application in the clinical and public health settings. The primary limitation of WHOQOL-100 was its length, which prevented it from being utilized in large epidemiological surveys where QOL is not the main variable. Therefore, a brief and more convenient WHOQOL-100 version, named WHOQOL-BREF, was developed as an alternative (1). The WHOQOL-BREF instrument consists of 26 questions to investigate the following domains: (1) psychological health, (2) environment, (3) social relationships, and (4) physical health. The 24 items represent the 24 dimensions of WHOQOL-100, the other two assess overall QOL status and overall health (10, 11). With fewer and more concentrated questions, the WHOQOL-BREF can reduce the response time of study participants while still being able to examine their QOL effectively (1).

Previous studies have adopted the WHOQOL-BREF to measure QOL of the general population (1, 10), specific general cohorts (e.g., adolescents, older adults, medical students, migrants, or women with childbirth) (12–17), or patients with specific conditions (18–21). The psychometric properties of this instrument have also been evaluated worldwide (12–17), most of which reported about acceptable reliability and validity of WHOQOL-BREF. However, inconsistency in the factorial structure *via* confirmatory factor analysis (CFA) still exists across different social settings. While some studies confirmed the validity of the WHO’s 4-model factors (22–24), others used alternative models with only three factors (in adolescents) (17), five factors (in the general population) (25), or even eight factors (in psychotic patients) (26). This inconsistency among WHOQOL-BREF models highlights a need for a comparison of different models and a guideline for employing this scale on specific populations in different settings.

In Vietnam, the validity of Vietnamese WHOQOL-BREF has been tested in community-dwelling older adults (27), people with hypertension (28), and HIV/AIDS patients under methadone maintenance therapy (29). However, only one study determined the factorial structure of this instrument (29) and none were conducted on general Vietnamese young adults. While young adults account for the majority of the population (30), there is a gap in WHOQOL-BREF validation studies on this population (17, 23). The characteristics of young adults are distinct from other age groups, as the transition from a dependent child to an independent adult, though unmeasurable, significantly influences the subjective perception of one’s QOL. Donald L. Patrick et al. developed the Youth Quality of Life Instrument (YQOL) indicating that internal factors such as self-efficacy or resilience and external factors such as environment and social relationships had a major role in shaping youths’ and adolescents’ QOL (31). The authors also found that when youths and adolescents suffered from negative conditions (e.g., diseases or mental impairment), they could still perceive good QOL if their internal and external perspectives could develop effective coping strategies (32). Given the complicated nature of the WHOQOL-BREF and its various associated factors, the purpose of our research is to explore the reliability and validity of the WHOQOL-BREF, version Vietnamese on Vietnamese young adults, as well as propose Vietnam adaptations for the model.

## Materials and methods

### Participants and study procedures

From April to June 2020, online cross-sectional research was applied in Vietnam to young adults. Participants had to fully meet the following criteria: (1) aged from 16 to 35; (2) lived in Vietnam, and (3) agreed to participate in the study. The snowball sampling technique was used to recruit participants from all provinces of Vietnam. A core group of Youth Union leaders in corporate organizations, companies, and public institutions was developed and invited to take the survey. The Vietnam Youth Union is the largest group of young adults in Vietnam with more than 7 million members from 14 to 35 years old. Participants also invited people in their networks and their peers to take this survey.

We applied a formula for a population mean to calculate the sample size of this study. The expected of Vietnamese Youth adults’ QOL score using WHOQOL-BREF instrument was 3.31 with standard deviation was 0.74 [according to a previous study using WHOQOL-BREF (33)], and the relative precision = 0.02. To compensate for the patients who might not answer the questionnaire completely or might end up refusing to participate, an additional 5% was added to the sample size. After calculating, the minimum sample size of this study was 505 participants. After data collection, 455 young people aged 16–35 from 38 of Vietnam’s 64 provinces completed the survey, with a response rate was 90.1%. The Youth Research Institute’s institutional review board authorized this study, which was carried out by the Helsinki Declaration guidelines (34).

### Measurement and instrument

We used Survey Monkey^1^ as the platform to develop an online survey for this study. This approach has the advantages of low cost, less time-consuming, user-friendly, and nationwide sample accessibility. Each survey should take about 10–15 min to complete. The information about socio-demographic characteristics, health status, and QOL *via* WHOQOL-BREF and the EuroQol-5 dimensions-5 levels (EQ-5D-5L) was collected through the structured questionnaire. Initially, the questionnaire was piloted with 5 young people to ensure that the multicultural value of the instruments remained after being translated into Vietnamese. The updated questionnaire was then posted to the online survey portal. The survey system was thoroughly tested before starting the data collection process to ensure that the substance of the questions was correct and that no technical difficulties arose. The following were the 39 items on the questionnaire:

*Health status and socio-demographic characteristics:* Participants reported their socio-demographic information including gender (male/female), education level (lower high school/above high school), marital status (single/married/others), area of residence (urban/suburban/rural/mountainous) and age. Data on health status were gathered by asking participants to describe any acute ailments they had in the preceding 4 weeks as well as any chronic conditions they had been diagnosed with in the previous 3 months.

*WHOQOL-BREF:* is a 26-item questionnaire to assess QOL, with the first two items being assessed and scored independently to assess overall perceptions of QoL (Q1), and satisfaction with general wellbeing (Q2). The remaining 24 items cover 24 aspects of four domains including the Environment domain (8 items), Physical health domain (7 items), Psychological health domain (6 items), and Social relationships domain (3 items). A 5-point Likert scale was used to rate these items, ranging from 1 (very poor/very dissatisfied/not at all/never) to 5 (very good/very satisfied/extremely/always). After reversing the raw scores of Q3, Q4, and Q26, the total scores of four domains were calculated to examine one’s perception of QOL in each corresponding domain. Higher scores indicate higher QOL (35). The Cronbach’s alpha of environment, physical, social relationship, and psychological domains were 0.884, 0.686, 0.764, and 0.713, respectively.

*EQ-5D-5L (5 items):* Data were obtained using the EQ-5D-5L profile and the EuroQol Visual Analogue Scale (EQ-VAS) (36). These instruments were widely accepted worldwide and were popular as measurement scales for the QOL of the Vietnamese population in general (37). EQ-5D-5L was applied to assess participants’ health problems in 5 aspects encompassing self-care, mobility, pain or discomfort, anxiety or depression, and usual activities. Each dimension was self-assessed on five levels: “no problems,” “slight problems,” “moderate problems,” “severe problems,” and “extreme problems.” Those who choose “slight problems” to “extreme problems” were grouped into the “Having problems” category, while others were grouped into the “No problem” category. The combination of responses of five domains could result in 3,125 health states. A Vietnamese cross-walk value set was used to convert the health states to corresponding utility scores (i.e., EQ-5D index) (38). The EQ-VAS was used to record the participants’ self-rated overall health on a vertical visual analog scale ranging from 0 “the worst possible” health to 100 “the best possible” health (39).

### Statistical analysis

STATA version 16 software was used to analyze the data. The significance level was set at 5% which is equivalent to a *p*-value ≤ 0.05. In a typical descriptive statistical study, the mean and standard deviation for quantitative data, as well as frequency and percentage for qualitative variables, were employed. The coefficients of skewness and kurtosis were examined. Floor and ceiling effects were detected when the percentage of participants picking the lowest or highest answer choice was more than 15%.

#### Reliability

The internal consistency reliability was examined by calculating Cronbach’s alpha, with an alpha value of 0.7 or above being considered acceptable (40). We also looked at, item-total correlation, item-item correlation, domain-domain correlation, and the domain’s Cronbach’s alpha if the item was removed.

#### Factorial structure

Based on the observed data, exploratory factor analysis (EFA) was utilized to contextualize the instrument’s structural model. The number of components was calculated using the scree plot and parallel analysis, as well as eigenvalues at 1.3 and the amount of variance explained. Items with a loading value ≥ 0.4 were considered to be included in the relevant component (41). Orthogonal Varimax rotation was used to assign items to appropriate domains.

Confirmation Factor Analysis (CFA) with means and variance adjusted weighted least squares (WLSMV) estimation method was used as recommended for ordinal scale (42). CFA was utilized to test whether the original model (i.e., 4 factors) of WHOQOL-BREF or the new model (i.e., 3 factors) could have a better explanation of the youths’ QOL. Measurement invariance of 3-factor model across priori-defined demographic groups regarding gender, age, marital status, education, living location, having acute/chronic conditions was tested by using four models as recommended (43, 44). We tested four models with different levels of measurement invariance: (1) configural invariance; (2) metric invariance; (3) strong (scalar) invariance; and (4) strict invariance. For configural invariance, we fitted the 3-factor model to every group by utilizing a single-group CFA method. This invariance revealed that the latent factors were similar across groups. For metric invariance, based on configural invariance, we equated all factor loadings of WHOQOL-BREF items and then tested if different items of latent factors had similar extent for all demographic groups. For strong (scalar) invariance, we equated the item thresholds to tested whether the model was fitted revealing no response bias across groups. To assess strict invariance, error variances from the scalar model were equated, and the fitted model indicated that error terms of items were similar across groups.

The model fit of observed data (with Satorra-Bentler adjustment for non-normality data) was then assessed using many models fit indicators with respective cut-offs, including (45): Relative Chi-square (χ 2/df) ≤ 3.0; Root Mean Square Error of Approximation (RMSEA) ≤ 0.08; Standardized Root Mean Square Residual (SRMR) ≤ 0.08 for a good fit and Comparative Fit Index (CFI) ≥ 0.9 for acceptable fit. The sample size in this study was sufficient for the measurement invariance (43, 44).

#### Convergent and divergent validity

The modified-WHOQOL-convergent BREF’s and divergent validity were investigated using Pearson’s correlation matrix between items-domain (46, 47). If the diagonal values were less than 0.4, there was inadequate convergent validity, and there was insufficient divergent validity if the off-diagonal values at each row were greater than the diagonal values. We created a Spearman’s correlation matrix between the scores of three domains, the general QOL satisfaction, general health satisfaction items, the EQ-5D index, and the EQ-VAS, to see if modified-WHOQOL-BREF had concurrent validity. The *t*-test was used to compare youth with acute symptoms in the last 4 weeks, chronic problems in the previous 3 months, and mobility, self-care, normal activities, pain/discomfort, and anxiety/depression concerns (EQ-5D domains). The difference in factor ratings between individuals with and without health concerns was measured using Cohen’s D effect size. To find substantial deviations, a value of 0.2 was employed (48).

### Ethical approval

This study was approved by the institutional review board of the Youth Research Institute and performed according to the Helsinki declaration guideline (49).

## Results

### Characteristics of respondents

Table 1 below shows sample characteristics, including health and socioeconomic demographics. The mean age of respondents was approximately 21.1 years (*SD* = 4.6). About 74.5% of respondents were female, 74.5% had a high school education or higher, 85% were still single, and 63% lived in suburban and urban areas. Respondents were then asked about their health status. Results showed that 16% of respondents reported that in the past 3 weeks they had suffered from chronic illnesses and that 43 percent of respondents reported that in the past 4 weeks they had suffered from acute symptoms. Half of the respondents reported that they were experiencing problems with depression and anxiety.

**TABLE 1:** Socio-demographic and health status characteristics of respondents.

Characteristics	Frequency (*n*)	Percentage (%)
Gender, female	339	74.5
Education, above the high school	339	74.5
**Living location**		
Urban	214	47.0
Suburban	74	16.3
Rural	157	34.5
Mountainous	10	2.2
**Marital status**		
Single	386	85.0
Married	56	12.3
Others	12	2.6
Having acute symptoms in the last 4 weeks	196	43.1
Having chronic conditions in the last 3 months	71	15.6
**EQ-5D-5L domains**		
Having problems with mobility	73	16.0
Having problems in self-care	20	4.4
Having problems in usual activities	62	13.6
Pain/Discomfort	136	29.9
Anxiety/Depression	218	47.9

	**Mean (SD)**	

Age, mean (SD)	21.1 (4.6)	
EQ-5D index, Mean (SD)	0.9 (0.1)	
EQ-VAS, Mean (SD)	86.3 (13.4)	

### Factorial structure of the World Health Organization’s quality of life instrument

**FIGURE 1:**
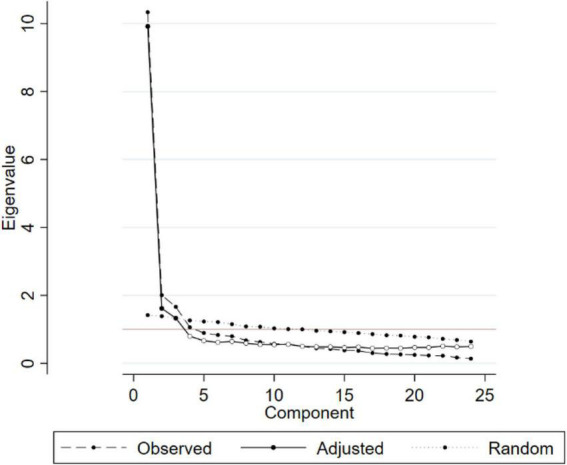
Scree parallel plot.

Figure 1 showed the scree plot and parallel analysis, which showed that the 3-factor model fitted with the current data.

Most community values are moderate. The Kaiser-Meyer-Olkin test indicated that the sample was sufficient for EFA with a score of 0.929. EFA is also useful to reconstruct WHOQOL-BREF based on Bartlett’s sphericity test (*P* < 0.01). Based on EFA and parallel screening, the three-factor model was the best method to evaluate WHOQOL-BREF. These factors comprised factor 1 “External life” (15 items), factor 2 “Internal life” (6 items), and factor 3 “Physical and mental health” (3 items) (Table 2). These factors could explain 92.9% of the total variance.

**TABLE 2:** Exploratory factor analysis for WHOQOL-BREF instrument.

Original domains	Variable	Factor 1: Satisfaction with external life	Factor 2: Satisfaction with internal life	Factor 3: Physical and mental health	Communality
Physical	Q3 Pain and discomfort*			0.6897	0.496
	Q4 Medication dependency*			0.7454	0.416
	Q10 Energy		0.5095		0.533
	Q15 Mobility	0.5198			0.590
	Q16 Sleep	0.6882			0.502
	Q17 Daily activities	0.7787			0.319
	Q18 Work capacity	0.7577			0.342
Psychological	Q5 Positive feelings		0.7537		0.380
	Q6 Spirituality		0.7558		0.403
	Q7 Concentration		0.564		0.487
	Q11 Body image	0.5547			0.603
	Q19 Self-esteem	0.6966			0.402
	Q26 Negative feelings*			0.4471	0.782
Social relationships	Q20 Personal relation	0.7527			0.347
	Q21 Sexual activity	0.5559			0.676
	Q22 Social support	0.7562			0.379
Environment	Q8 Safety		0.6146		0.383
	Q9 Healthy home environment		0.5182		0.543
	Q12 Financial resources	0.4807			0.625
	Q13 Information	0.6032			0.581
	Q14 Recreation	0.6499			0.527
	Q23 Physical environment	0.8054			0.298
	Q24 Health care	0.7973			0.330
	Q25 Transport	0.7641			0.389

*Reverse-coded.

Table 3 shows the difference in the internal structure and model fit indices of the 4-factor model and 3-factor model of the WHOQOL-BREF. Results of the overall goodness of fit (χ2/df), as well as other fit indicators (RMSE, CFI, and SRMR), suggested that the 3-factor model is better than the theoretical 4-factor model.

**TABLE 3:** Goodness-of-fit indices of 3-factor model and 4-factor model.

Domain	3-factor model	4-factor model
χ^2^ (df)	485.24 (237)	595.73 (221)
χ^2^/df	2.047	2.696
RMSE	0.065	0.08
CFI	0.931	0.902
SRMR	0.054	0.058

df, degree of freedom; RMSE, Root Mean Square Error of Approximation; CFI, Comparative Fit Index; SRMR, Standardized Root Mean Square Residual.

### Data quality and reliability

Table 4 shows the results of using the descriptive method for the 24 categories of WHOQOL-BREF. Each category has a range of 1–5 points, of which 23/24 items had ceiling effects except body image (Q11) and 23/24 items revealed no floor effects except pain and discomfort (Q3). The kurtosis coefficient varied from 2.0 to 4.0, and the skewness coefficient varied from –1.0 to 1.0. The results showed that respondents had a favorable perception of their QOL -based on the standard deviation and the mean deviation of each item’s scores. The modified-WHOQOL-reliability BREF’s is also shown in Table 2. Factor 1 “External life” and factor 2 “Internal life” had outstanding (0.931) and good internal consistency, respectively (0.864), respectively, while the internal consistency of factor 3 “Physical and mental health” was nearly acceptable (0.690). The majority of the items in respective components had medium correlation values with other items (*r* > 0.4).

**TABLE 4:** Basic descriptions and reliability of WHOQOL-BREF instrument.

Items	Mean (SD)	Skewness	Kurtosis	Floor (%)	Ceiling (%)	Item-total correlation	Cronbach’s alpha if an item deleted
Q1 General QOL	3.9 (0.7)	–0.13	2.46	0.0	21.3		
Q2 General health	3.8 (0.9)	–0.33	3.14	1.5	24.0		
**Factor 1: external life**							
Q11 Body image	3.4 (1.0)	–0.22	3.23	4.2	15.0	0.419	0.938
Q12 Financial resources	3.4 (1.2)	–0.17	2.26	7.0	23.3	0.425	0.935
Q13 Information	3.7 (0.9)	–0.3	2.8	1.3	21.8	0.426	0.934
Q14 Recreation	3.5 (1.0)	–0.18	2.33	2.0	20.7	0.415	0.933
Q15 Mobility	4.2 (0.9)	–0.92	3.50	1.3	43.7	0.431	0.936
Q16 Sleep	3.7 (1.0)	–0.30	2.82	2.6	22.6	0.418	0.933
Q17 Daily activities	3.8 (0.9)	–0.32	3.09	1.5	24.0	0.415	0.930
Q18 Work capacity	3.8 (0.8)	–0.26	3.19	1.3	20.0	0.420	0.931
Q19 Self-esteem	3.8 (0.9)	–0.16	2.79	1.1	22.9	0.421	0.931
Q20 Personal relation	3.7 (0.8)	–0.13	2.89	1.1	20.7	0.418	0.930
Q21 Sexual activity	3.7 (1.0)	–0.54	3.33	4.8	24.6	0.429	0.937
Q22 Social support	3.7 (0.8)	0.01	2.65	0.9	21.1	0.421	0.931
Q23 Physical environment	3.7 (0.8)	–0.09	3.03	1.1	17.8	0.418	0.930
Q24 Health care	3.7 (0.8)	–0.12	3.20	1.5	17.8	0.419	0.931
Q25 Transport	3.7 (0.9)	–0.26	3.06	2.0	22.4	0.417	0.931
**Factor 2: internal life**							
Q5 Positive feelings	3.8 (1.0)	–0.74	3.67	4.2	23.1	0.433	0.825
Q6 Spirituality	3.9 (1.0)	–0.92	3.69	4.2	32.1	0.437	0.833
Q7 Concentration	3.6 (0.9)	–0.41	3.56	2.9	15.6	0.444	0.823
Q8 Safety	3.7 (0.9)	–0.40	3.34	2.6	20.2	0.423	0.811
Q9 Home environment	3.6 (0.9)	–0.33	3.03	2.4	17.6	0.448	0.830
Q10 Energy	4.2 (0.8)	–0.94	3.29	1.8	24.6	0.455	0.840
**Factor 3: physical and mental health**							
Q3 Pain and discomfort*	2.7 (1.3)	0.12	2.00	18.9	24.0	0.431	0.474
Q4 Medication dependency*	2.1 (1.2)	0.79	2.60	7.0	44.6	0.470	0.502
Q26 Negative feelings*	2.6 (1.1)	0.30	2.54	11.0	19.3	0.944	0.759

**Domain scores**							**Cronbach’s alpha**

External life (15–75)	55.5 (10.1)	0.11	3.24	0.2	7.2		0.931
Internal life (6–30)	22.7 (4.3)	–0.51	3.82	0.7	7.0		0.864
Physical and mental health (3–15)	10.6 (2.8)	–0.59	3.10	3.3	7.5		0.690

*Reverse-coded.

**FIGURE 2:**
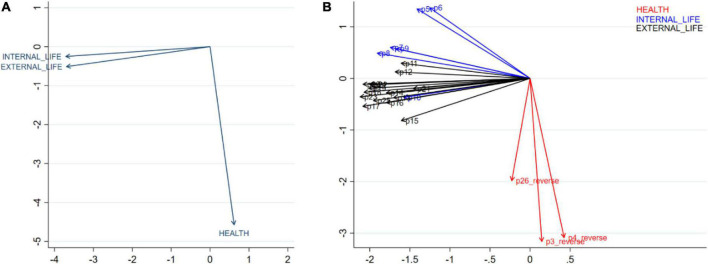
Correlation between domains **(A)** and items **(B)**.

Item-item and factor-factor correlations are shown in Figures 2A,B below. Factor 1 “External life” and factor 2 “Internal life” are more likely to be correlated, but both factors showed a non-correlation with factor 3 “Physical and mental health.” Similarly, the items in “External life” and “Internal life” were more likely to be correlated with others while items Q3-Pain and discomfort, Q4-Medication dependency, and Q26-Negative feelings seem to have within-group correlation but not to be correlated with items in other factors.

### Validity

**FIGURE 3:**
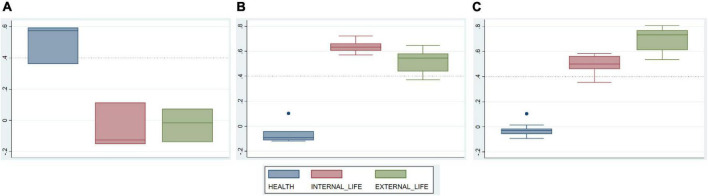
Correlations between items of each domain and other domain scores. **(A)** Items of domain “physical and mental health”; **(B)** items of domain “internal life”; **(C)** items of domain “external life.”

Boxplots in Figure 3 and results in Table 3 illustrate the correlations between items and factor scores, which reflect the convergent and divergent validity of the modified WHOQOL-BREF instrument. The item-domain correlations in factor 1 (green box) and factor 2 (pink box) were similar which means the similarity between the two factors. Meanwhile, the item-domain correlation in factor 3 (blue box) seemed to be significantly different from the other two factors, suggesting better divergent validity.

Table 5 reveals an acceptable convergent validity. There are 23/24 items (excluding Q26 Negative sentiments) that had correlation coefficients larger than 0.4 with their corresponding component scores. Furthermore, each item correlates better with its component scores than the other factor scores, showing good divergent validity. Only one item (Q26-Negative feelings) had a low correlation coefficient with its factor (*r* = 0.362).

**TABLE 5:** Correlation matrix between items and domain scores to assess convergent and divergent validity.

	Factor 1: external life	Factor 2: internal life	Factor 3: physical and mental health
**Factor 1: external life**			
Q11 Body image	**0.545**	0.536	–0.057
Q12 Financial resources	**0.611**	0.479	–0.071
Q13 Information	**0.635**	0.45	–0.015
Q14 Recreation	**0.676**	0.461	–0.034
Q15 Mobility	**0.572**	0.492	0.105
Q16 Sleep	**0.674**	0.438	–0.023
Q17 Daily activities	**0.788**	0.555	0.014
Q18 Work capacity	**0.769**	0.569	–0.029
Q19 Self-esteem	**0.733**	0.584	–0.016
Q20 Personal relation	**0.780**	0.571	–0.047
Q21 Sexual activity	**0.535**	0.354	–0.049
Q22 Social support	**0.754**	0.515	–0.066
Q23 Physical environment	**0.806**	0.563	–0.021
Q24 Health care	**0.769**	0.5	–0.092
Q25 Transport	**0.747**	0.488	–0.015
**Factor 2: internal life**			
Q5 Positive feelings	0.439	**0.644**	–0.118
Q6 Spirituality	0.371	**0.605**	–0.113
Q7 Concentration	0.582	**0.663**	–0.102
Q8 Safety	0.647	**0.722**	–0.04
Q9 Home environment	0.557	**0.621**	–0.081
Q10 Energy	0.535	**0.570**	0.102
**Factor 3: physical and mental health**			
Q3 Pain and discomfort*	–0.016	–0.127	**0.594**
Q4 Medication dependency*	–0.139	–0.152	**0.575**
Q26 Negative feelings*	0.074	0.115	**0.362**

*Reverse-coded. Bold values mean the best value correlation matrix between items and domain score.

Table 6 reveals that factor 3 “Physical and mental health” exhibited the lowest correlations with two general items, the EQ-5D score and the EQ-VAS (rh0 < 0.2). The factor 1 score and the general QOL item had the highest correlation coefficient (rh0 = 0.5968, *p* < p0.05), followed by the factor 1 score and the general health item (rh0 = 0.5895, *p* < 0.05).

**TABLE 6:** Spearman’s correlation matrix with the WHOQOL-BREF instrument’s general quality of life satisfaction and general health satisfaction questions, EQ-5D index, and EQ-VAS to assess concurrent validity.

Domains/Items	EQ-5D index	EQ-VAS	Q1 general QOL	Q2 general health	Factor 1: external life	Factor 2: internal life	Factor 3: physical and mental health
EQ-5D index	1	0.3415*	0.3865*	0.4673*	0.3935*	0.2924*	0.1750*
EQ-VAS	0.3415*	1	0.3976*	0.5176*	0.3384*	0.3399*	0.0960*
Q1 general QOL	0.3865*	0.3976*	1	0.6718*	0.5968*	0.5160*	0.1237*
Q2 general health	0.4673*	0.5176*	0.6718*	1	0.5895*	0.5431*	0.1471*
Factor 1: external life	0.3935*	0.3384*	0.5968*	0.5895*	1	0.7239*	0.0931*
Factor 2: internal life	0.2924*	0.3399*	0.5160*	0.5431*	0.7239*	1	0.0367
Factor 3: physical and mental health	0.1750*	0.0960*	0.1237*	0.1471*	0.0931*	0.0367	1

*p-value < 0.05.

The adjusted instrument’s discriminant validity was tested, and the findings are shown in Table 7. Mobility problems, self-care problems, chronic illness (3 months ago) and acute symptoms (4 weeks ago) had significantly lower scores on the “external life” factor. out” with a *P*-value less than 0.05. The Cohen’s D effect sizes between those with and without conditions ranged from 0.33 (95% CI = 0.07–0.58) to 0.78 (95% CI = 0.58–0.97). Meanwhile, the score of factor 2 “Internal life” was significantly lower among those with problems in mobility, usual activities, pain/discomfort, and anxiety or depression compared to people without problems. Individuals with problems in every domain of EQ-5D-5L instruments had a significantly lower score in factor 3 “Physical and mental health” than those without problems.

**TABLE 7:** Discriminant validity of 3-factor model of WHOQOL-BREF instrument.

Characteristics	External life			Internal life			Physical and mental health		
			
	Mean (SD)	*P*-value	Effect size Cohen’s D (95% CI)	Mean (SD)	*P*-value	Effect size Cohen’s D (95% CI)	Mean (SD)	*P*-value	Effect size Cohen’s D (95% CI)
**Having acute symptoms in the last 4 weeks**									
No (*n* = 259)	57.2 (10.7)	<0.01	0.39 (0.20–0.57)*	22.9 (4.6)	0.20	0.11 (–0.08 to 0.29)	10.6 (3.1)	0.35	0.00 (–0.18 to 0.19)
Yes (*n* = 197)	53.4 (8.7)			22.5 (3.8)			10.6 (2.4)		
**Having chronic conditions in the last 3 months**									
No (*n* = 385)	56.0 (10.3)	0.03	0.33 (0.07–0.58)*	22.8 (4.4)	0.36	0.12 (–0.13 to 0.38)	10.6 (2.9)	0.12	0.12 (–0.13 to 0.38)
Yes (*n* = 71)	52.8 (8.3)			22.3 (4.0)			10.3 (2.2)		
**EQ-5D-5L domains**									
**Having problems with mobility**									
No (*n* = 383)	56.4 (9.9)	<0.01	0.57 (0.32–0.83)*	23.1 (4.0)	<0.01	0.60 (0.34–0.85)*	10.7 (2.9)	<0.01	0.26 (0.01–0.51)*
Yes (*n* = 73)	50.8 (9.4)			20.6 (5.1)			10.0 (2.4)		
**Having problems in self-care**									
No (n = 436)	55.7 (9.8)	0.52	0.29 (–0.15 to 0.74)	22.8 (4.2)	0.12	0.52 (0.07–0.96)*	10.7 (2.8)	0.02	0.50 (0.05–0.95)*
Yes (n = 20)	52.7 (13.8)			20.6 (5.9)			9.3 (2.9)		
**Having problems in usual activities**									
No (n = 394)	56.3 (9.8)	<0.01	0.54 (0.27–0.81)*	23.1 (4.2)	<0.01	0.58 (0.31–0.85)*	10.7 (2.9)	<0.01	0.35 (0.08–0.62)*
Yes (n = 62)	50.9 (10.2)			20.6 (4.6)			9.7 (2.5)		
**Pain/Discomfort**									
No (*n* = 319)	57.2 (10.0)	<0.01	0.58 (0.38–0.78)*	23.1 (4.5)	<0.01	0.28 (0.08–0.48)*	10.8 (3.0)	<0.01	0.25 (0.05–0.45)*
Yes (*n* = 137)	51.6 (8.9)			21.9 (3.7)			10.1 (2.3)		
**Anxiety/Depression**									
No (*n* = 237)	59.0 (10.0)	<0.01	0.78 (0.58–0.97)*	23.7 (4.7)	<0.01	0.47 (0.29–0.66)*	10.7 (3.3)	0.02	0.11 (–0.08 to 0.29)
Yes (*n* = 219)	51.7 (8.6)			21.7 (3.6)			10.4 (2.2)		

*p < 0.05.

Table 8 illustrate the measurement invariance of WHOQOL-Bref in youth population. Results showed that all models were fitted with CFI and RMSEA in acceptable range, even after adding constraints. These models demonstrate that the 3-factor model had the same pattern of factor loadings, item threshold and residual variances across groups regarding gender, age, education, marital status, living location, having acute symptoms and having chronic diseases.

**TABLE 8:** Model fit and nested model comparisons for multiple group CFA analyses.

Group	Invariance	Δx^2^(df)	Δx^2^/df	CFI	RMSEA (90%CI)	Δ x^2^(Δ df)	ΔCFI	Δ RMSEA
Gender	Configural	744.630 (498)	1.495	0.896	0.047 (0.040; 0.054)		–	–
	Metric	688.024 (519)	1.326	0.929	0.038 (0.030; 0.045)	56.606 (21)	0.033	0.009
	Strong	711.255 (540)	1.317	0.928	0.037 (0.029; 0.045)	33.375 (21)	0.001	0.001
	Strict	744.367 (564)	1.320	0.924	0.037 (0.030; 0.045)	33.112 (24)	0.004	0.000
Education	Configural	722.682 (498)	1.451	0.919	0.045 (0.037; 0.052)		–	–
	Metric	688.066 (519)	1.326	0.939	0.038 (0.030; 0.045)	34.616 (21)	0.020	0.007
	Strong	713.837 (540)	1.322	0.937	0.038 (0.030; 0.035)	25.771 (21)	0.002	0.000
	Strict	739.073 (564)	1.310	0.937	0.037 (0.029; 0.044)	25.236 (24)	0.000	0.001
Marital status	Configural	660.884 (498)	1.327	0.934	0.038 (0.030; 0.046)		–	–
	Metric	601.698 (519)	1.159	0.967	0.027 (0.015; 0.035)	59.186 (21)	0.033	0.006
	Strong	627.627 (540)	1.162	0.965	0.027 (0.016; 0.036)	25.929 (21)	0.002	0.000
	Strict	655.739 (564)	1.163	0.963	0.027 (0.016; 0.035)	28.112 (24)	0.002	0.000
Age group	Configural	974.683 (747)	1.305	0.923	0.045 (0.037; 0.053)		–	–
	Metric	958.260 (789)	1.215	0.943	0.038 (0.029; 0.046)	16.423 (42)	0.020	0.007
	Strong	1019.779 (831)	1.227	0.936	0.039 (0.030; 0.047)	61.519 (42)	0.007	0.001
	Strict	1085.746 (897)	1.210	0.93	0.040 (0.031; 0.048)	65.967 (48)	0.006	0.001
Location	Configural	726.268 (498)	1.458	0.919	0.045 (0.038; 0.052)		–	–
	Metric	710.496 (519)	1.369	0.932	0.040 (0.033; 0.048)	15.772 (21)	0.013	0.005
	Strong	734.051 (540)	1.359	0.931	0.040 (0.032; 0.047)	23.555 (21)	0.001	0.001
	Strict	757.850 (564)	1.344	0.931	0.039 (0.031; 0.046)	23.799 (24)	0.000	0.001
Acute symptoms	Configural	756.857 (498)	1.520	0.907	0.048 (0.041; 0.055)		–	–
	Metric	757.599 (519)	1.460	0.914	0.045 (0.038; 0.052)	0.742 (21)	0.007	0.003
	Strong	782.806 (540)	1.450	0.912	0.045 (0.038; 0.051)	25.207 (21)	0.002	0.000
	Strict	810.681 (564)	1.437	0.911	0.044 (0.037; 0.051)	27.875 (24)	0.001	0.001
Chronic diseases	Configural	675.399 (498)	1.356	0.931	0.040 (0.032; 0.047)		–	–
	Metric	634.207 (519)	1.222	0.955	0.031 (0.022; 0.039)	41.192 (21)	0.024	0.008
	Strong	655.481 (540)	1.214	0.955	0.031 (0.021; 0.039)	21.274 (21)	0.000	0.000
	Strict	679.896 (564)	1.205	0.955	0.030 (0.021; 0.038)	24.415 (24)	0.000	0.001

## Discussion

This study is a systematic examination of the psychometric properties of the Vietnamese version of WHOQOL-BREF for measuring the QOL of young adults in Vietnam. Overall, current findings supported the use of WHOQOL-BREF in this population group as indicated by acceptable internal consistency and moderate validity of the instrument. Moreover, the EFA and CFA proposed a new factorial structure model (3 factors) with better model fits than the original one (4 factors), suggesting a new theoretical and culture-sensitive approach to assessing the QOL of young adults in Vietnam.

Regarding data distribution, results demonstrate that responses were skewed to higher scores in all items, indicating high ceiling effects. This result was in line with previous studies performed on general populations such as older adults in the community, couples, or residents in urban areas (22, 24, 50). Ceiling effects were recorded in all items except body image. This exception might imply that the WHOQOL-BREF instrument is not able to capture the responsiveness to change of young people with high scale scores (22). Interestingly, our findings indicated that people could still have a high general QOL score even if their general health satisfaction scores were, which suggested that health is only a minor concern in determining the QOL in the young adult population.

Results of the EFA indicated that Vietnamese young adults assessed QOL more easily under three factors instead of four factors as in the original WHOQOL-BREF. The first dimension was “External life” which employed most of the items from “Social relationships” and “Environment” and several items in “Physical health” and “Psychological health” in the original scoring system. Another dimension was “Internal life,” which was related to personal traits such as enjoyment, spirituality/meaning life, concentration, life energy, and environmental conditions that they feel safe or healthy. The last dimension was “Physical and mental health” which was associated directly with pain/discomfort, medication use, or negative feelings (e.g., depression, anxiety, or despair). This result is expectable as previous studies also indicated that internal and external factors had a major role in the QOL perception of young adults (32). Findings from discriminant validity analysis indicated that in the general Vietnamese young adults population, the existence of diseases or illnesses only affected the external life domain and no statistically significant difference was found in internal life or physical and mental health domains. This result suggested that Vietnamese young adults were more likely to consider the influences of diseases on their daily activities and social interactions rather than on health itself. Our regression results confirmed this hypothesis, as the “External life” and “Internal life” domains were more correlated to the general QOL, general health, and overall QOL than physical and mental health.

In terms of statistics, the results of two CFA models suggested that the 3-factor model showed better model fit indices compared to the theoretical 4-factor model of the WHOQOL-BREF. In the existing literature, although the psychometric properties of the WHOQOL-BREF were widely field-examined in a diversity of cultures and groups (1, 10, 19), it is clear they vary across populations. Although some authors have attempted to use CFA to confirm the 4-factor structure of the WHOQOL-BREF (19, 24–26), many failed to replicate the theoretical model across different population groups (17, 25, 26). One potential explanation for this trend might be that some items could not fully represent the domains in that they were postulated (22, 51). For example, Skevington et al. suggested that the “safety” item should be allocated in the psychological health domain rather than the environment domain (10). Findings in convergent and divergent validity also demonstrated that correlations between items and their new respective domains were higher than correlations with other domains. These findings suggest a need for further studies to reassess the structure of WHOQOL-BREF.

Considering internal consistency scores of WHOQOL-BREF domains, the reliability of both 4-factor and 3-factor models was both acceptable. In the 4-factor model, the environment domain had the highest Cronbach’s alpha, which was consistent with previous studies (17, 52, 53). However, the advantage of the 3-factor model as suggested by EFA was that its screen parallel analysis had better estimates than the 4-factor model, in which “External life” had the highest Cronbach’s alpha (0.931). The third domain, “Physical and mental health,” after removing the item “Negative feelings,” might improve the internal consistency from 0.690 to 0.759. Similar to previous studies (10, 50), this item was preserved for further analysis.

To our knowledge, this is the first attempt in examining the measurement properties of WHOQOL-BREF among Vietnamese young adults. The results of this study suggested several practical and research implications. First, this study highlighted the need to carefully evaluate the dimensionality of the WHOQOL-BREF in different population groups considering their unique perceptions of QOL definitions. Second, as the applicability of WHOQOL-BREF has been confirmed, it should be used more widely in Vietnam considering its ability to cover a wide range of QOL’s facets. The WHOQOL-BREF instrument can also be useful in routine auditing for young adults-related health and social services (10), as well as their development progress. The last and most important implication of WHOQOL-BREF is its possibility for 100-point transformation, which can be used as a cross-culturally comparable index. Cross-cultural validation should be among the main concerns of future research to develop appropriate factorial structures for different young adult groups such as those with disabilities or chronic diseases. For instance, a previous study found that “acceptance of disability” was an important component when defining the QOL of young adults with chronic disabilities (54).

The strength of this study lies in the diversity of its samples. We were able to include young adults from various locations in Vietnam, which increased the representativeness of the results. However, certain limitations exist. First, as our sample was recruited online, it could not assess the QOL of youths without Internet access. Second, because the majority of our data was gathered through self-reporting, they might be subject to recall and response bias. Third, we could not examine test-retest reliability because all participants’ information such as name or contact details (e.g., email, mobile phone, etc.) was not collected to ensure survey privacy, which prevented us from resending the survey to evaluate its reliability. Nevertheless, our study was able to confirm the validity of the scale as well as highlight several implications for its adaptation in Vietnam.

## Conclusion

This validation study provides insights into the characteristics of QOL among young adults in Vietnam. While the theoretical model of WHO can be utilized for global comparisons, a new local model, which includes cross-cultural adaptations, should be developed to fully evaluate the progress of public health interventions for promoting young adults’ QOL.

## Data availability statement

The original contributions presented in this study are included in the article/supplementary material, further inquiries can be directed to the corresponding author.

## Ethics statement

This study was approved by the Institutional Review Board of the Youth Research Institute and performed according to the Helsinki declaration guideline. The patients/participants provided their written informed consent to participate in this study.

## Author contributions

LN, CN, and CL: conceptualization. LV, LN, and CN: data curation. LV and GV: formal analysis. CL, RH, and CH: investigation. GV, RH, and CH: supervision. LN, GV, RH, and CH: writing—original draft. LV, LN, CN, and CL: writing—review and editing. All authors contributed to the article and approved the submitted version.

## Footnotes

1
https://www.surveymonkey.com/
